# Accessing physical activity among young adults attending a university: the role of sex, race/ethnicity, technology use, and sleep

**DOI:** 10.1186/s12889-017-4757-y

**Published:** 2017-09-18

**Authors:** Samuel D. Towne, Marcia G. Ory, Matthew Lee Smith, S. Camille Peres, Adam W. Pickens, Ranjana K. Mehta, Mark Benden

**Affiliations:** 1 Department of Health Promotion and Community Health Sciences, School of Public Health, Texas A&M University, 1266 TAMU, College Station, TX 77843-1266 USA; 2 Center for Population Health and Aging, School of Public Health, Texas A&M University, College Station, TX 77843-1266 USA; 3 Center for Population Health and Aging & Department of Environmental and Occupational Health, School of Public Health, Texas A&M University, 1266 TAMU, College Station, TX 77843-1266 USA; 4 Department of Health Promotion and Behavior, College of Public Health, The University of Georgia, 330 River Road, 315 Ramsey Center, Athens, GA 30602 USA; 5 Texas A&M Ergonomics Center & Department of Environmental and Occupational Health, School of Public Health, Texas A&M University, 1266 TAMU, College Station, TX 77843-1266 USA

**Keywords:** Physical activity, Young adults, Sociodemographic, College students, Technology, Sleep

## Abstract

**Background:**

Identifying factors associated with recommended physical activity (PA) levels are critical in efforts to combat the obesity epidemic and related comorbidities.

**Methods:**

We conducted cross-sectional analyses of college students (*n* = 490) enrolled in a large southern state university in October of 2014. Our aim was to identify sociodemographic characteristics, technology use, and sleep patterns among college students and their independent relationship to recommended PA. An online survey was sent to all enrolled students. Logistic regression predicted achieving recommended ≥150 min per week of moderate-vigorous PA (MVPA) versus not (≤149 min MVPA).

**Results:**

Approximately 69% of study participants were males, 18% were Hispanic, and more than half (60%) were within the normal body mass index (12% were obese). The average age of students was 21 years. On a daily average, individuals used smartphones most often (nearly 4.4 h), followed by laptops at 4.0 h, desktops at 1.2 h, and tablets at 0.6 h. The mean number of hours individuals reported sleeping was 6.7. Sociodemographic factors associated with reporting ≥150 min of MVPA included being male (OR = 4.0, 95% CI 2.2–7.1) versus female, being non-Hispanic White (OR = 1.8, CI 1.1–3.2) versus being a member of minority race group. Behavioral factors associated with reporting ≥150 min of MVPA included technology use (being moderate-heavy (OR = 2.3, CI 1.1–4.8) or heavy (OR = 3.4, CI 1.6–7.5) users of technology), and receiving low-moderate (OR = 1.9, 1.01–3.7) levels of sleep versus the lowest level of sleep.

**Conclusions:**

In the current study, minority status and being female were the strongest sociodemographic factors associated with inadequate PA levels, while high technology use (primarily driven by smartphone use) were associated with recommended PA levels. Identifying factors associated with being physically active will allow for targeted interventions to improve the health of young adults.

## Background

Identifying factors associated with engagement in recommended physical activity (PA) levels is critical in efforts to combat the obesity epidemic and related disease [1]. The Centers for Disease Control and Prevention (CDC) recommend adult individuals participate in at least 150 min of moderate or 75 min of vigorous physical activity (or a combination) per week [2]. This level of PA was selected because studies show it can prevent obesity, an array of chronic conditions (e.g. lower risk of type 2 diabetes, cardiovascular disease), certain cancers (e.g. colon, breast), improve general well-being (e.g. improved sleep quality, reduced depression) [3], and increased cognitive capacity [4–6]. It is important to identify factors associated with physical activity across the life-course as healthy habits may be formed or changed at any life stage. However, many health behaviors develop early in life, which is one reason it is important to identify factors associated with physical activity in young adults [7, 8].

According to the National Center for Education Statistics (NCES), over 20 million students were expected to attend universities in the US in 2015, up 4.9 million since 2000 [9]. This growth in the population means more and more individuals will be classified as college graduates or having some college experience. This gives rise to the need to understand factors associated with living healthy lifestyles which can be measured by health-related outcomes (e.g., health behaviors). Education has already been shown to be associated with physical activity, with a higher percentage of college graduates reported as engaging in regular physical activity compared to those without a high school education [10]. Among the estimated 20 million students expected to attend universities in the US in 2015, an estimated 39.9% were between the ages of 18–24 years of age [9]. Thus, university students make up a large number young adults in the US. However, only approximately half of on-campus college students report meeting recommended moderate to vigorous physical activity levels [11]. Further, over 60% of US adults between the ages of 20–39 years were either overweight or obese, while nearly a third were obese (data from 2011/2012) [12]. Therefore, identifying factors associated with physical activity among this rapidly growing group may have implications for millions of individuals throughout the US both now and in the future.

### Technology use

Several behavioral factors may affect physical activity among young adults. For example, the growing use of technology has been suggested as a possible contributor to lower physical activity levels among the global community including in the United States [13]. Investigations are currently underway that examine the mental and physical impacts on college students using technology displays and input devices such as smartphones, which have become the pervasive means of communicating and performing school tasks [14]. In the US, 86% of those between the ages of 18–29 owned smartphones in 2015, followed by computers (78%) and tablet computers (50%) [15]. However, only limited research on how technology use affects health behaviors, namely physical activity is available. For example, the link between mobile phone use and sedentary behavior among college students is theorized to disrupt routine and consistent physical activity, which can ultimately lead to reduced cardiorespiratory fitness levels [16]. Further, a recent study showed a positive relationship between mobile phone use and sedentary behavior among college students [17]. However, other evidence suggest mixed results among male and female university students, where a negative relationship was detected with sedentary technology use (e.g. computer use) and physical activity among males, but no relationship was detected among females [18]. Thus, research that identifies the relationship between utilization of technological devices and health behaviors, namely physical activity, is critical and timely given the rapid development of new technologies, increased use of multiple types of technological devices (e.g. smartphones, tables, and computers) among young adults, and the limited evidence to date.

### Sleep

There are many health-related factors that may impact physical activity. For example, sleep is necessary for maintaining normal functioning, yet sleep deprivation may be more prevalent among college or university students [19]. Factors associated with inadequate sleep among college students may include what one study called poor sleep hygiene (e.g. consumption of alcohol and caffeine, technology use prior to sleep) and sleep disorders [19]. Some evidence also suggests that short-term sleep loss is associated with decreased levels of physical activity [20]. Thus, understanding the relationship between sleep and physical activity among young adults in university settings may be necessary to gain a more complete picture into factors associated with physical activity among this population.

### Sociodemographic characteristics

Physical activity levels may also be influenced by sex and race/ethnicity. One study of several US counties assessing physical activity from 2000 to 2011 found higher levels of physical activity among males as compared to females, yet increases in physical activity were higher among females during the same timeline [21]. Thus, sex may play an important role in physical activity among young adults in university settings. Racial and ethnic differences may also be associated with physical activity. In the US, racial and ethnic disparities existed with regard to physical activity among adults [10]. Among both men and women, non-Hispanic White adults had higher regular physical activity than all other groups [10]. Thus, racial and ethnic variation may be important in identifying factors associated with physical activity.

#### Aims

The aims of the current study were to: 1) identify physical activity levels among a sample of college students and college graduates currently enrolled in college classes (i.e., graduate students); and 2) examine the relationship between sociodemographic (e.g. sex, race/ethnicity) and behavioral factors (e.g., sleep, technology use) associated with physical activity, namely meeting the recommended threshold of moderate-to-vigorous minutes per week. This research is important because of the known impact of physical activity on health and well-being during later life and that college is a time where many young adults begin to establish their long-term health habits [7, 8, 22].

## Methods

### Population & setting

Undergraduate and graduate students enrolled in a science field were included. Students were drawn from those enrolled in a single department at a large southern tier-1 university in October of 2014. This baseline assessment was part of a larger participatory ergonomics program that lasted a full school year during Fall 2014 and Spring 2015 semesters. It is important to note that the data used in this study were collected prior to the introduction of any training or intervention about ergonomics.

### Design

We conducted a cross-sectional analyses of survey responses distributed to college students during the start of the Fall semester. As part of administrative procedures, an online survey (created in Qualtrics, Qualtrics LLC) was sent via email to all enrolled students. Survey responses were completed as part of their school registration process and as such no incentives were used for participation. Thus, all enrolled students attending the following semester were targeted for inclusion. There were 490 completed survey responses among initiated survey responses. The response rate was approximately 87.6% (*n* = 429) for undergraduate students and 48.4% (*n* = 61) for graduate students.

### Measures

The survey was a combination of measures used in multiple existing surveys and also created by ergonomists on the study team. For example, the Behavioral Risk Factor Surveillance System (BRFSS) was used to assess demographic information pertaining to race, ethnicity, and sex. Physical activity items were *informed* by the World Health Organization’s Global physical activity questionnaire analysis guide [23] and others [24]. Exposure and use of technology survey items were designed by ergonomist on the study team.

### Outcomes

#### Physical activity

Physical activity was defined as the average number of minutes of moderate to vigorous physical activity (MVPA) per week calculated from self-reported survey responses. Survey items asking the number of days and minutes of moderate and separately vigorous physical activity per week were asked. Sample survey items for moderate physical activity included: *On average, how many days a week do you engage in physical activity that is moderate (*e.g. *brisk walking)? On the days you are moderately active, on average how many minutes are you active each day? (these can be accumulated throughout the day).* Moderate minutes and vigorous minutes were combined using a multiplier of 2.0 for vigorous as instructed by the Centers for Disease Control and Prevention’s publication: A Data Users Guide to the BRFSS Physical Activity Questions [25]. This provided a single variable of MVPA [2]. Given we were interested in factors associated with meeting the recommended level of physical activity, we categorized MVPA into ≥150 min of moderate-vigorous PA (MVPA) versus <149 min of MVPA per week.

### Independent variables

#### Technology-related behavior: Use and exposure of technology

Use and exposure of technology were primary variables of interest. This was defined as the type of device (i.e. smartphone, tablet, laptop, desktop computer) used and the exposure (duration of use). Use was calculated based on the following questions: *What types of interactive technology devices do you use most often?* Options consisted of the following devices: smartphone, tablet, laptop, and desktop computer. Respondents were also asked to rank the devices in order on the screen. The device listed first was assigned a 1, while the device listed last was assigned a 4.

Exposure was calculated based on the following questions: *On average, how many hours do you spend interacting with the following device(s) each day?* Time was measured separately for smartphone, tablet, laptop, and desktop computer. We included greater detail on technology use in order to provide greater insight on the type of device used most given the interaction with a smartphone or tablet may not be as restrictive as using a desktop. For example, one is likely at a desk with a chair while using a desktop versus potentially mobile (e.g., walking) while using a smartphone or tablet.

Summary statistics were used for descriptive analyses by device type. Further analyses assessed technology use classified into quartiles based on the number of hours of use per day on average. Use of any device (i.e. smartphone, tablet, laptop, desktop) was combined to create a variable coded as any technology use measured in hours. This variable was then separated into quartiles described as low, low-moderate, moderate-high, and high. The decision to use the quartiles as cut-points, based on the distribution of our data, was driven by the relatively limited data for comparisons with a similar sample of survey respondents. Thus, this provides a relative comparison between lower and higher technology users among our sample.

#### Health-related behavior: sleep

Average hours (per night) spent sleeping was separated into quartiles, and described as low, low-moderate, moderate-high, and high. In addition to being quartiles splits, these cut-points matched with the sleep guidelines highlighted on the CDC’s website (https://www.cdc.gov/sleep/about_sleep/how_much_sleep.html) and Watson et al. [26] where adults aged 18–60 years are recommended to get 7 or more hours of sleep per night. Thus, the cut-points (≤6 h, 7 h, 8 h, and more than 8 h) allowed us to assess comparisons of those not meeting the recommendation relative to differing degrees of meeting this guideline (i.e., 7 h, 8 h, and more than 8 h). This was taken from a single survey item: *On average, how many hours of sleep do you get in a 24-h period? Think about the time you actually spend sleeping or napping, not just the amount of sleep you think you should get.*

##### Control variables

Self-reported race was treated as White, Black, Asian, Native Hawaiian or other Pacific Islander, American Indian or Alaska Native. Given the distribution of racial groups, we coded race as White or minority status. Ethnicity was coded as Hispanic or non-Hispanic. For multivariate analyses, the combination of race and ethnicity included non-Hispanic White versus racial and ethnic minority individuals. Sex was coded as male or female and age was treated as a continuous variable. Body Mass Index (BMI) was calculated from self-reported height and weight. BMI was included as a categorical covariate in adjusted analyses and classified into underweight (BMI <18.5), normal weight (BMI ≥18.5 and <25), overweight (BMI ≥25 and <30), and obese (BMI ≥30).

### Statistical analyses

SAS 9.4 (Cary, NC) was used in all analyses. Bivariate and multivariate analyses were conducted and chi square tests were used to assess differences in sample distribution. Logistic regression predicted achieving ≥150 min of moderate-vigorous PA (MVPA) versus not (≤149 min MVPA). The fully adjusted model included BMI category, sex, the combination of race/ethnicity (non-Hispanic White versus racial and ethnic minority), age, sleep (quartiles), and technology use (quartiles). The interaction between sleep and technology use was initially tested in addition to this full model. Given the interaction was not significant, we report findings without the interaction present.

## Results

### Sample characteristics

Overall, the average age of students was 21 years (range 18–46 years). The majority (85%) of respondents met or exceeded the recommended physical activity guidelines versus 15% who failed to report having at least 150 min of MVPA per week. Regarding sleep behaviors, the mean number of hours individuals reported sleeping was 6.7 (median 7.0).

The distribution of the sample by selected characteristics was presented in Table 1. Respondents were predominately male (69%), non-Hispanic (82%), and White (84%). In terms of the combination of race and ethnicity, 68% were non-Hispanic White leaving 32% that identified as a racial or ethnic minority. Further, the majority of the sample was within the normal BMI range (60%), with 12% reporting classified as obese.

**Table 1:** Respondent distribution on selected demographic and health-related behaviors and outcomes

	Total	Physical Activity				Technology Use								Sleep							
		Meeting PA Guidelines		Failing to meet PA Guidelines		Low		Low-moderate		Moderate-high		High		Low		Low-moderate		Moderate-high		High	
		≥150 min per week		≤149 min per week		Lower quartile (≤ 6 h)		> Lower quartile and ≤ median (> 6 h and ≤8 h)		> Median and ≤upper quartile (>8 h and <12 h)		> Upper quartile (≥12 h)		Lower quartile (≤ 6 h)		> Lower quartile and ≤median (> 6 h and ≤7 h)		> Median and ≤upper quartile (>7 and ≤8 h)		> Upper quartile (>8 h)	
	N	N	%	N	%	N	%	N	%	N	%	N	%	N	%	N	%	N	%	N	%
Sex																					
Male	339	303	89.38%	36	10.62%	131	38.64%	66	19.47%	7	22.12%	67	19.76%	137	40.41%	96	28.32%	77	22.71%	29	8.55%
Female	151	114	75.50%	37	24.50%	24	15.89%	28	18.54%	35	23.18%	64	42.38%	60	39.74%	55	36.42%	26	17.22%	10	6.62%
Ethnicity																					
Hispanic	90	73	81.11%	17	18.89%	15	16.67%	12	13.33%	29	32.22%	34	37.78%	46	51.11%	23	25.56%	15	16.67%	6	6.67%
Non-Hispanic	400	344	86.00%	56	14.00%	140	35.00%	82	20.50%	81	20.25%	97	24.25%	151	37.75%	128	32.00%	88	22.00%	33	8.25%
Race																					
White	409	352	86.06%	57	13.94%	134	32.76%	80	19.56%	97	23.72%	98	23.96%	164	40.10%	128	31.30%	87	21.27%	30	7.33%
Non-White	81	65	80.25%	16	19.75%	21	25.93%	14	17.28%	13	16.05%	33	40.74%	33	40.74%	23	28.40%	16	19.75%	9	11.11%
Race/Ethnicity																					
Non-Hispanic White	333	290	87.09%	43	12.91%	122	36.64%	70	21.02%	71	21.32%	70	21.02%	124	37.24%	110	33.03%	73	21.92%	26	7.81%
Racial/ethnic Minority	157	127	80.89%	30	19.11%	33	21.02%	24	15.29%	39	24.84%	61	38.85%	73	46.50%	41	26.11%	30	19.11%	13	8.28%
BMI																					
Underweight	23	18	78.26%	5	21.74%	8	34.78%	3	13.04%	5	21.74%	7	30.43%	7	30.43%	11	47.83%	2	8.70%	3	13.04%
Normal weight	292	248	84.93%	44	15.07%	91	31.16%	62	21.23%	70	23.97%	69	23.63%	116	39.73%	91	31.16%	63	21.58%	22	7.53%
Overweight	119	103	86.55%	16	13.45%	44	36.97%	22	18.49%	23	19.33%	30	25.21%	49	41.18%	39	32.77%	23	19.33%	8	6.72%
Obese	56	48	85.71%	8	14.29%	12	21.43%	7	12.50%	12	21.43%	25	44.64%	25	44.64%	10	17.86%	15	26.79%	6	10.71%

### Descriptive comparisons

#### Physical activity

Overall, among comparisons within sex, ethnicity, and race, we find that the majority of respondents reported meeting the physical activity guidelines with 89% of males and 76% of females, 81% of Hispanic individuals, and 86% of White individuals reporting at least 150 min of moderate-to-vigorous physical activity per week. In terms of the combination of race and ethnicity, we find that 87% of non-Hispanic White individuals and 81% of racial/ethnic minority individuals reported at least 150 min of moderate-to-vigorous physical activity per week. Further, we find that 78% of underweight individuals, 85% of normal weight individuals, 87% of overweight individuals, and 86% of obese individuals reported at least 150 min of moderate-to-vigorous physical activity per week. In terms of the relative comparisons between groups, the difference in proportions indicate the magnitude of differences.

#### Technology use

Overall, approximately 32% of respondents used technology for at or lower than 6 h per day (classified as the *lowest use category of technology*) on average. Overall, approximately 27% of respondents used technology for 12 or more hours per day (classified as the *highest use category of technology*).

#### Sleep

Overall, 40% of participants were classified as having at or lower than 6 h of sleep per day (classified as the *lowest level of sleep*) on average. Overall, 8% of participants were classified as having at least 9 h of sleep per day (classified as the *highest level of sleep*) on average.

##### Bivariate analyses with physical activity, technology use, and sleep

When testing for relationships between demographic characteristics and physical activity, we found males (versus females) were more likely (OR = 2.7, 95% CI 1.6–4.4) to meet the recommended guidelines versus not. When testing for relationships between demographic characteristics and technology use, we found: males (versus females) were less likely (OR = 0.3, 95% CI 0.2–0.5) to have the highest quartile of technology use (≥12 h) versus fewer hours of technology use (<12 h); Hispanic individuals (versus non-Hispanic individuals) were more likely (OR = 1.8, 95% CI 1.1–2.9) to have the highest quartile of technology use (≥12 h) versus fewer hours of technology use (<12 h); White individuals (versus non-White individuals) were less likely (OR = 0.4, 95% CI 0.3–0.7) to have the highest quartile of technology use (≥12 h) versus fewer hours of technology use (<12 h); non-Hispanic White individuals (versus racial/ethnic minority individuals) were less likely (OR = 0.4, 95% CI 0.3–0.6) to have the highest quartile of technology use (≥12 h) versus fewer hours of technology use (<12 h); normal weight individuals (versus obese individuals) were less likely (OR = 0.4, 95% CI 0.2–0.7) to have the highest quartile of technology use (≥12 h) versus fewer hours of technology use (<12 h); overweight individuals (versus obese individuals) were less likely (OR = 0.4, 95% CI 0.2–0.8) to have the highest quartile of technology use (≥12 h) versus fewer hours of technology use (<12 h). When testing for relationships between demographic characteristics and sleep, we found Hispanic individuals (versus non-Hispanic individuals) were more likely (OR = 1.7, 95% CI 1.04–2.6) to have the lowest quartile of sleep (≤6 h) versus more hours of sleep (>6 h) (Table 2).

**Table 2:** Bivariate analyses with physical activity, technology use, and sleep by select demographics

	Meeting PA Guidelines			Upper quartile of technology (≥12 h) versus other (<12 h)			Lower quartile of sleep (≤ 6 h) versus other (>6 h)		
	Odds Ratio	95% Confidence Interval		Odds Ratio	95% Confidence Interval		Odds Ratio	95% Confidence Interval	
Sex									
Male versus female	2.683^a^	1.626	4.426	0.336^a^	0.222	0.510	1.070	0.725	1.580
Ethnicity									
Hispanic versus Non-Hispanic	0.679	0.378	1.220	1.819^a^	1.125	2.942	1.645^a^	1.043	2.594
Race									
White versus Non-White	1.451	0.787	2.675	0.441^a^	0.269	0.722	0.992	0.612	1.608
Race/Ethnicity									
Non-Hispanic White versus Racial/ethnic Minority	1.584	0.957	2.622	0.419^a^	0.277	0.633	0.713	0.486	1.044
BMI Category									
Underweight versus Obese	0.712	0.211	2.402	0.527	0.189	1.467	0.491	0.177	1.366
Normal weight versus Obese	1.038	0.476	2.262	0.400^a^	0.222	0.720	0.777	0.439	1.376
Overweight versus Obese	1.207	0.498	2.926	0.431^a^	0.221	0.841	0.835	0.442	1.577

^a^ indicates significance (*p* < .05)

##### Physical activity by technology use and sleep

Table 3 presents results of physical activity by technology use and sleep. *Among those who met the physical activity guidelines*: Those individuals classified as having at or lower than 6 h of technology use per day on average were the largest group at 31% (*n* = 128) followed by 28% (*n* = 115) among participants classified as having more than 8 h of technology use per day on average. *Among those who did not met the physical activity guidelines*: Those individuals classified as having at or lower than 6 h of technology use per day on average were the largest group at 37% (*n* = 27).

**Table 3:** Technology Use and Sleep by Physical Activity

		Physical Activity	
		Meeting PA Guidelines	Failing to meet PA Guidelines
		≥150 min per week	≤149 min per week
Technology use			
Low	Lower quartile (≤ 6 h)	128	27
Low-moderate	> Lower quartile and ≤ median (> 6 h and ≤8 h)	79	15
Moderate-high	> Median and ≤upper quartile (>8 h and <12 h)	95	15
High	> Upper quartile (≥12 h)	115	16
Sleep			
Low	Lower quartile (≤ 6 h)	159	38
Low-moderate	> Lower quartile and ≤median (> 6 h and ≤7 h)	132	19
Moderate-high	> Median and ≤upper quartile (>7 and ≤8 h)	92	11
High	> Upper quartile (>8 h)	34	5

*Among those who met the physical activity guidelines*: Those individuals classified as having at or lower than 6 h of sleep per day on average were the largest group at 38% (*n* = 159) followed by 32% (*n* = 132) among participants who were classified as having 7 h of sleep per day on average. *Among those who did not met the physical activity guidelines*: Those individuals classified as having at or lower than 6 h of sleep per day on average were the largest group at 52%.

### Technology use and exposure

Table 4 presents more detail on technology use and exposure. Overall, 94% of respondents reported smartphone use making it the highest category of technology use followed by laptops (91%), desktops (34%), and tablets (27%). On average, individuals used smartphones 4.4 h per day, followed by laptops at 4.0 h, desktops at 1.2 h, and tablets at 0.6 h. When users were asked to estimate the percent of their total device interaction time by activity (subjective to the time spent on other devices), users reported 49.9% of the time was for school or class, followed by recreation (33.3%) and work (9.2%) on average.

**Table 4:** Technology use and exposure (*n* = 490)

	Smartphone		Tablet		Laptop		Desktop	
Use	N	%	N	%	N	%	N	%
Technology with any use	459	93.67	131	26.74	447	91.25	166	33.88
Technology by ranking of use^a^								
1st	305	64.21	16	3.46	130	27.37	23	4.87
2nd	128	26.95	45	9.72	253	53.26	47	9.96
3rd	23	4.84	149	32.18	74	15.58	224	47.46
4th	19	4.00	253	54.64	18	3.79	178	37.71
Exposure	Mean (Standard Deviation)	Median	Mean (Standard Deviation)	Median	Mean (Standard Deviation)	Median	Mean (Standard Deviation)	Median
Average number of hours of use per day	4.42 (+/−3.79)	3.0	0.57 (+/−1.12)	0.0	3.97 (+/−2.92)	4.0	1.23 (+/−1.86)	1.0

^a^ Percentage based on percent within each device category

### Multivariate analyses

Multivariate analyses (see Table 5) accounts for the simultaneous inclusion of the following predictors in the model: BMI category, sex, the combination of race/ethnicity (non-Hispanic White versus racial and ethnic minority), age, sleep (quartiles), and technology use (quartiles). Multivariate analyses were based on 417 individuals meeting the physical activity guidelines versus 73 who did not. Sociodemographic factors associated with reporting ≥150 min of MVPA included being male (OR = 4.0, 95% CI 2.2–7.1) versus female, being non-Hispanic White (OR = 1.8, CI 1.1–3.2) versus identifying as a racial or ethnic minority individual. Being moderate-heavy (OR = 2.3, CI 1.1–4.8) or heavy (OR = 3.4, CI 1.6–7.5) users of technology versus low users was associated with reporting ≥150 min of MVPA. Receiving low-moderate levels (OR = 1.9, 1.01–3.7) versus the lowest levels of sleep was associated with reporting ≥150 min of MVPA.

**Table 5:** Multivariate analyses assessing the likelihood of meeting the recommended physical activity guidelines (≥150 min per week of moderate-to-vigorous physical activity)

	Odds Ratio	95% Confidence Interval	
BMI Category			
Underweight versus Obese	0.853	0.218	3.339
Normal weight versus Obese	1.025	0.426	2.465
Overweight versus Obese	1.182	0.447	3.127
Sex			
Male versus female	3.972^a^	2.224	7.095
Race			
Non-Hispanic White versus Racial or Ethnic Minority	1.844^a^	1.055	3.222
Technology use by quartile			
Low-moderate versus low	1.513	0.720	3.179
Moderate-high versus low	2.277^a^	1.071	4.844
High versus low	3.418^a^	1.555	7.509
Sleep by quartile			
Low-moderate versus low	1.915^a^	1.006	3.645
Moderate-high versus low	1.933	0.917	4.077
High versus low	1.818	0.626	5.278

Fully adjusted analyses: Meeting MVPA guideline *n* = 417; failing to meet MVPA guideline *n* = 73

^a^ indicates significance (*p* < .05)

## Discussion

This study provided a unique glimpse into the sociodemographic, health- and technology-related factors associated with meeting recommended PA levels among a sample of young adult college students in the science field. Findings confirmed that college students are heavy technology users [15], and that females [21] and minority [10] participants were less likely to be engaged in adequate PA levels than their respective peers. Further, that high technology use may differ by demographic characteristics (e.g., lower for normal weight and overweight individuals versus obese individuals).

Participants in this sample engaged in more PA than others their age across the nation [27] and their high technology use (primarily driven by smartphone use) was associated with meeting recommended levels of PA. Compared to other literature about the use of technology by adults and college students [28–30], our findings indicate an increase in total daily technology use was associated with a higher likelihood of meeting the physical activity guidelines. Further, this is driven primarily by smartphone use, which is likely attributed to the proliferation of apps and social media [31]. The widespread availability and affordability of smartphones in the United States (in addition to apps and other technological devices) has increased their use exponentially [31]. Further, the nature of a mobile device means these students can use technology while on the move; operating a motor vehicle, walking/biking, or exercising in the gym.

As such, while the common paradigm is that increased technology and screen time are positively associated with sedentary behavior, it may be that mobile technologies are not stifling PA and can actually promote PA. Indeed, biomedical innovations have enabled a recent influx of technological applications (particularly smartphones) to monitor, model, and promote physical activity [32–34]. In comparing previous research, it can be seen that Melton et al., (2014) found that technology use among college students (*n* = 591) in 2012 was just 852 min per week [35]. Thus, while this study is similar to the extent that it is restricted to college students, our study recorded much higher average time engaged in technology use. This may highlight a growth in technology use, albeit the methods and sample were not identical and neither study was national in scope making any extrapolation limited.

College education is very technology-inclusive, as indicated by about half of our study participants reporting ‘school or class purposes’ as their primary use of technology. While students are often required to use technology and the internet to complete most school-related tasks (e.g. sending email, submitting assignments, reading assigned content), nearly all web-based services are now “mobile-friendly.” The traditional computer interfaces have been modified for use on smartphones, which drastically diminishes the need to complete tasks while physically sitting at a computer station or office (or at one place at one time). In the growing world of multi-tasking young adults, this means school-related tasks could potentially be accomplished while performing other activities such as physical activity. Further, when asked about how the technology devices were used, over one-third of study participants reported ‘recreation’ and another nearly 10% reported ‘work.’

However, information about the types of activities were not collected. For example, depending on the type of recreation (e.g. hiking, biking, walking) or work (e.g. working in a retail store or warehouse requiring constant movement), the PA levels reported by participants may vary. While these data are neither definitive nor widely generalizable, these technology-PA relationships warrant further exploration.

We also found that receiving recommended levels (except 7 h) of sleep (approximately 8 h or more) was associated with recommended PA, a finding reported in the literature [36]. Additionally, the median number of hours individuals reported sleeping was 7, which is relatively consistent with other reports among young adults [37]. Because the importance of sleep is gaining recognition as it is viewed as a health determinant, symptom of other morbidities, and health outcomes [38], its association with PA is not surprising. In fact, PA is known to improve mood [39], reduce stress [39], and enhance sleep quality [40]. Therefore, the relationship between sleep and PA in this cross-sectional study may reflect that those who are engaging in recommended PA levels are getting adequate sleep.

### Limitations

Given the cross-sectional nature of this study, we were unable to draw causal inferences about the relationships observed. Thus, we present associations between variables of interest and our outcomes. In future studies, we recommend that data be collected at multiple time points to identify trends over time. Our study findings may not be widely generalizable to other populations, given the sample was pulled from a single department at one university and has a very limited demographic range. Further, given the sample was pulled from a science field, the results may not be generalizable to non-science majors. Given large national studies with our outcomes among college students have yet to be carried out, the degree to which our sample varies from that of the nation is difficult to specify. As noted earlier, Melton et al., (2014) identified different results for technology use (i.e., much lower use) among college students in their study, albeit from 2012 data. As noted previously, participants in this study reported high levels of PA, which may not represent other college students. Further, PA was based solely on the recommended aerobic recommendations and did not assess strength training, although students may have included both types of activities in their reporting of PA. The lack of significant findings for BMI as related to PA in the fully adjusted model may be due to limited sample sizes across BMI categories and the self-reported nature of the variables (i.e. height and weight), and the fact that we were only interested in comparing PA in relation to the recommended guidelines in the current study and not overall PA. It should also be noted that these data were self-reported, and we were unable to determine the true accuracy of time spent engaging in PA, using technology, and/or sleeping. While this is a limitation of the current study, it highlights the importance of including more objective measures in future studies to more accurately document behaviors of interest (e.g. personal activity trackers, smartphone apps documenting use, home sleep tests). Lastly, the decision to use cut-points rather than treating variables as continuous is not without limitations. Using cut-points may allow for meaningful relative comparisons. That said, treating variables as continuous may be a useful default without proper justification for cut-points. Thus, the implications of these findings should be taken in light of these limitations.

## Conclusions

The study provides a unique and timely perspective of factors associated with PA among college students. Identifying new and evolving factors associated with being physically active will allow for targeted interventions to improve the health of young adults, especially those in technology-intensive fields in a university setting. Future studies should examine technology use in analyses to identify patterns of use in relation to physical activity as well as other health-related behaviors.

The growing prevalence of technology and its utilization will continue to shape individuals’ health-related behaviors. Identifying factors associated with the use of technology and health-related behaviors is critical if we are to continue to develop and implement innovative, timely, and adaptive health-related interventions. However, whether the true potential of technology in the form of hand-held devices can actually improve physical activity among young adults remains to be seen.

## Abbreviations

BMI: Body Mass Index
MVPA: Moderate-to-Vigorous Physical Activity
PA: physical activity
